# Supplementary material for: Effects of Gluten-Free Diet in Non-Celiac Hashimoto’s Thyroiditis: A Systematic Review and Meta-Analysis
Source: Nutrients. 2025 Oct 31;17(21):3437. doi: 10.3390/nu17213437 (PMC12609533; doi:10.3390/nu17213437)
Supplement: Supplementary file 1 [file nutrients-17-03437-s001.zip › nutrients-3746443-supplementary.pdf]

SUPPLEMENTARY MATERIAL

_Supplementary material S1 - PRISMA Checklist 2020

Section and Topic	Item #	Checklist item	Location where item is reported
**TITLE**			
Title	1	Identify the report as a systematic review.	2-3
**ABSTRACT**			
Abstract	2	See the PRISMA 2020 for Abstracts checklist.	25-44
**INTRODUCTION**			
Rationale	3	Describe the rationale for the review in the context of existing knowledge.	47-93
Objectives	4	Provide an explicit statement of the objective(s) or question(s) the review addresses.	89-93
**METHODS**			
Eligibility criteria	5	Specify the inclusion and exclusion criteria for the review and how studies were grouped for the syntheses.	105-126 (Table 1)
Information sources	6	Specify all databases, registers, websites, organisations, reference lists and other sources searched or consulted to identify studies. Specify the date when each source was last searched or consulted.	128-142
Search strategy	7	Present the full search strategies for all databases, registers and websites, including any filters and limits used.	Supplementary material – S2 and S3.
Selection process	8	Specify the methods used to decide whether a study met the inclusion criteria of the review, including how many reviewers screened each record and each report retrieved, whether they worked independently, and if applicable, details of automation tools used in the process.	154-168
Data collection process	9	Specify the methods used to collect data from reports, including how many reviewers collected data from each report, whether they worked independently, any processes for obtaining or confirming data from study investigators, and if applicable, details of automation tools used in the process.	154-168
Data items	10a	List and define all outcomes for which data were sought. Specify whether all results that were compatible with each outcome domain in each study were sought (e.g. for all measures, time points, analyses), and if not, the methods used to decide which results to collect.	170-189

	10b	List and define all other variables for which data were sought (e.g. participant and intervention characteristics, funding sources). Describe any assumptions made about any missing or unclear information.	226-231
Study risk of bias assessment	11	Specify the methods used to assess risk of bias in the included studies, including details of the tool(s) used, how many reviewers assessed each study and whether they worked independently, and if applicable, details of automation tools used in the process.	191-207
Effect measures	12	Specify for each outcome the effect measure(s) (e.g. risk ratio, mean difference) used in the synthesis or presentation of results.	215-224
Synthesis methods	13a	Describe the processes used to decide which studies were eligible for each synthesis (e.g. tabulating the study intervention characteristics and comparing against the planned groups for each synthesis (item #5)).	209-213
	13b	Describe any methods required to prepare the data for presentation or synthesis, such as handling of missing summary statistics, or data conversions.	226-231
	13c	Describe any methods used to tabulate or visually display results of individual studies and syntheses.	209-213
	13d	Describe any methods used to synthesize results and provide a rationale for the choice(s). If meta-analysis was performed, describe the model(s), method(s) to identify the presence and extent of statistical heterogeneity, and software package(s) used.	215-224
	13e	Describe any methods used to explore possible causes of heterogeneity among study results (e.g. subgroup analysis, metaregression).	233-242
	13f	Describe any sensitivity analyses conducted to assess robustness of the synthesized results.	244-246
Reporting bias assessment	14	Describe any methods used to assess risk of bias due to missing results in a synthesis (arising from reporting biases).	248-251
Certainty assessment	15	Describe any methods used to assess certainty (or confidence) in the body of evidence for an outcome.	253-265
**RESULTS**			
Study selection	16a	Describe the results of the search and selection process, from the number of records identified in the search to the number of studies included in the review, ideally using a flow diagram.	268-280; Fig 1
	16b	Cite studies that might appear to meet the inclusion criteria, but which were excluded, and explain why they were excluded.	Supplementary material – S4
Study characteristics	17	Cite each included study and present its characteristics.	295-308; Table 2
Risk of bias in studies	18	Present assessments of risk of bias for each included study.	310-438
Results of individual studies	19	For all outcomes, present, for each study: (a) summary statistics for each group (where appropriate) and (b) an effect estimate and its precision (e.g. confidence/credible interval), ideally using structured tables or plots.	310-438<br>Figures 2-9

Results of syntheses	20a	For each synthesis, briefly summarise the characteristics and risk of bias among contributing studies.	310-438
	20b	Present results of all statistical syntheses conducted. If meta-analysis was done, present for each the summary estimate and its precision (e.g. confidence/credible interval) and measures of statistical heterogeneity. If comparing groups, describe the direction of the effect.	310-438<br>Figures 2-9
	20c	Present results of all investigations of possible causes of heterogeneity among study results.	310-438<br>Figures 2-9
	20d	Present results of all sensitivity analyses conducted to assess the robustness of the synthesized results.	440-442
Reporting biases	21	Present assessments of risk of bias due to missing results (arising from reporting biases) for each synthesis assessed.	444-447
Certainty of evidence	22	Present assessments of certainty (or confidence) in the body of evidence for each outcome assessed.	310-438<br>Supplementary material – S6
**DISCUSSION**			
Discussion	23a	Provide a general interpretation of the results in the context of other evidence.	458-656
	23b	Discuss any limitations of the evidence included in the review.	649-651
	23c	Discuss any limitations of the review processes used.	652-653
	23d	Discuss implications of the results for practice, policy, and future research.	653-656
**OTHER INFORMATION**			
Registration and protocol	24a	Provide registration information for the review, including register name and registration number, or state that the review was not registered.	29
	24b	Indicate where the review protocol can be accessed, or state that a protocol was not prepared.	97-99
	24c	Describe and explain any amendments to information provided at registration or in the protocol.	449-453
Support	25	Describe sources of financial or non-financial support for the review, and the role of the funders or sponsors in the review.	679-682
Competing interests	26	Declare any competing interests of review authors.	691
Availability of data, code and other materials	27	Report which of the following are publicly available and where they can be found: template data collection forms; data extracted from included studies; data used for all analyses; analytic code; any other materials used in the review.	685-686

From: Page MJ, McKenzie JE, Bossuyt PM, Boutron I, Hoffmann TC, Mulrow CD, et al. The PRISMA 2020 statement: an updated guideline for reporting systematic reviews. BMJ 2021;372:n71. doi: 10.1136/bmj.n71. This work is licensed under CC BY 4.0. To view a copy of this license, visit https://creativecommons.org/licenses/by/4.0/

_Supplementary material S2 - Search strategy, February 4th, 2025

Cochrane Library –
Results: 21

#1 ('gluten free diet':ti,ab,kw OR 'gluten-free':ti,ab,kw OR 'gluten deprivation':ti,ab,kw OR 'gluten restriction':ti,ab,kw OR 'gluten withdrawal':ti,ab,kw OR 'gluten elimination':ti,ab,kw OR 'gluten exclusion':ti,ab,kw OR 'gluten avoidance':ti,ab,kw OR 'gluten removal':ti,ab,kw OR

#2 MeSH descriptor: [Diet, Gluten-Free] explode all trees

#3 #1 OR #2

#4 (('thyroid disease':ti,ab,kw OR 'autoimmune thyroiditis':ti,ab,kw OR 'thyroiditis':ti,ab,kw OR 'thyroid inflammation':ti,ab,kw OR 'hashimoto':ti,ab,kw OR 'hashimoto`s thyroiditis':ti,ab,kw OR 'hashimoto`s disease':ti,ab,kw OR 'chronic lymphocytic thyroiditis':ti,ab,kw OR 'autoimmune thyroiditis':ti,ab,kw OR 'tsh':ti,ab,kw OR 'thyroid stimulating hormone':ti,ab,kw OR 'thyrotropin':ti,ab,kw OR 'thyrotropic hormone':ti,ab,kw OR 'tsh hormone':ti,ab,kw OR 'ft4':ti,ab,kw OR 'free thyroxine':ti,ab,kw OR 'free t4':ti,ab,kw OR 'free thyroxine level':ti,ab,kw OR 'serum free t4':ti,ab,kw OR 'free thyroxine concentration':ti,ab,kw OR 'anti-thyroiantibodies':ti,ab,kw OR 'thyroid autoantibodies':ti,ab,kw OR 'anti-thyroid peroxidase antibodies':ti,ab,kw OR 'anti-thyroglobulin antibodies':ti,ab,kw OR 'thyroid antibodies':ti,ab,kw OR 'tgab':ti,ab,kw OR 'anti-thyroglobulin antibodies':ti,ab,kw OR 'anti-tg':ti,ab,kw OR 'thyroglobulin antibodies':ti,ab,kw OR 'anti-tg antibodies':ti,ab,kw OR 'tg autoantibodies':ti,ab,kw OR 'tpoab':ti,ab,kw OR 'anti-thyroid peroxidase antibodies':ti,ab,kw OR 'anti-tpo':ti,ab,kw OR 'thyroid peroxidase antibodies':ti,ab,kw OR 'anti-tpo antibodies':ti,ab,kw OR 'tpo autoantibodies':ti,ab,kw))

#5 MeSH descriptor: [Thyroid Diseases] explode all trees

#6 #4 OR #5

#7 #3 AND #6

EMBASE (Elsevier)

Results: 83

#1

(('gluten free diet'/exp OR 'gluten-free':ti,ab,kw OR 'gluten deprivation':ti,ab,kw OR 'gluten restriction':ti,ab,kw OR 'gluten withdrawal':ti,ab,kw OR 'gluten elimination':ti,ab,kw OR 'gluten exclusion':ti,ab,kw OR 'gluten avoidance':ti,ab,kw OR 'gluten removal':ti,ab,kw))

#2
(('thyroid disease'/exp OR 'autoimmune thyroiditis'/exp OR 'thyroiditis':ti,ab,kw OR 'thyroid inflammation':ti,ab,kw OR 'hashimoto':ti,ab,kw OR 'hashimoto`s thyroiditis':ti,ab,kw OR 'hashimoto`s disease':ti,ab,kw OR 'chronic lymphocytic thyroiditis':ti,ab,kw OR 'autoimmune thyroiditis':ti,ab,kw OR 'tsh':ti,ab,kw OR 'thyroid stimulating hormone':ti,ab,kw OR 'thyrotropin':ti,ab,kw OR 'thyrotropic hormone':ti,ab,kw OR 'tsh hormone':ti,ab,kw OR 'ft4':ti,ab,kw OR 'free thyroxine':ti,ab,kw OR 'free t4':ti,ab,kw OR 'free thyroxine level':ti,ab,kw OR 'serum free t4':ti,ab,kw OR 'free thyroxine concentration':ti,ab,kw OR 'anti-thyroid antibodies':ti,ab,kw OR 'thyroid autoantibodies':ti,ab,kw OR 'anti-thyroid peroxidase antibodies':ti,ab,kw OR 'anti-thyroglobulin antibodies':ti,ab,kw OR 'thyroid antibodies' OR 'tgab':ti,ab,kw OR 'anti-thyroglobulin antibodies':ti,ab,kw OR 'anti-tg':ti,ab,kw OR 'thyroglobulin antibodies':ti,ab,kw OR 'anti-tg antibodies':ti,ab,kw OR 'tg autoantibodies':ti,ab,kw OR 'tpoab':ti,ab,kw OR 'anti-thyroid peroxidase antibodies':ti,ab,kw OR 'anti-tpo':ti,ab,kw OR 'thyroid peroxidase antibodies':ti,ab,kw OR 'anti-tpo antibodies':ti,ab,kw OR 'tpo autoantibodies':ti,ab,kw))

#3
‘randomized controlled trial’/exp OR ‘controlled clinical trial’/de OR random*:ti,ab,tt OR ‘randomization’/de OR ‘intermethod comparison’/de OR placebo:ti,ab,tt ‘double blind procedure’/de OR (parallel NEXT/1 group*):ti,ab,tt OR (crossover:ti,ab,tt OR ‘cross over’:ti,ab,tt) OR (compare:ti,tt OR compared:ti,tt OR comparison:ti,tt) OR ((evaluated:ab OR evaluate:ab OR evaluating:ab OR assessed:ab OR assess:ab) AND (compare:ab OR compared:ab OR comparing:ab OR comparison:ab)) OR (open NEXT/1 label):ti,ab,tt OR ((double OR single OR doubly OR singly) NEXT/1 (blind OR blinded OR blindly)):ti,ab,tt OR ((assign* OR match OR matched OR allocation) NEAR/6 (alternate OR group OR groups OR intervention OR interventions OR patient OR patients OR subject OR subjects OR participant OR participants)):ti,ab,tt OR (assigned:ti,ab,tt OR allocated:ti,ab,tt) OR (controlled NEAR/8 (study OR design OR trial)):ti,ab,tt OR (volunteer:ti,ab,tt OR volunteers:ti,ab,tt) OR ‘human experiment’/de OR trial:ti,tt OR (((random* NEXT/1 sampl* NEAR/8 (‘cross section*’ OR questionnaire* OR survey OR surveys OR database or databases)):ti,ab,tt) NOT (‘comparative study’/de OR ‘controlled study’/de OR ‘randomised controlled’:ti,ab,tt OR ‘randomized controlled’:ti,ab,tt OR ‘randomly assigned’:ti,ab,tt)) OR (‘cross-sectional study’/de NOT (‘randomized controlled trial’/exp OR ‘controlled clinical trial’/de OR ‘controlled study’/de OR ‘randomised controlled’:ti,ab,tt OR ‘randomized controlled’:ti,ab,tt OR ‘control group’:ti,ab,tt OR ‘control groups’:ti,ab,tt)) OR (‘case control*’:ti,ab,tt AND random*:ti,ab,tt NOT (‘randomised controlled’:ti,ab,tt OR ‘randomized controlled’:ti,ab,tt)) OR ‘random field*’:ti,ab,tt OR (‘random cluster’ NEAR/4 sampl*):ti,ab,tt
#4 #1 AND #2 AND #3

LILAC’s (BVS)

Results: 1

("gluten free diet" OR "gluten-free" OR "gluten deprivation" OR "gluten restriction" OR "gluten withdrawal" OR "gluten elimination" OR "gluten exclusion" OR "gluten avoidance" OR "gluten removal") AND ("thyroid disease" OR "autoimmune thyroiditis" OR "thyroiditis" OR "thyroid inflammation" OR "hashimoto" OR "hashimoto`s thyroiditis" OR "hashimoto`s disease" OR "chronic lymphocytic thyroiditis" OR "autoimmune thyroiditis" OR "tsh" OR "thyroid stimulating hormone" OR "thyrotropin" OR "thyrotropic hormone" OR "tsh hormone" OR "ft4" OR "free thyroxine" OR "free t4" OR "free thyroxine level" OR "serum free t4" OR "free thyroxine concentration" OR "anti-thyroid antibodies" OR "thyroid autoantibodies" OR "anti-thyroid peroxidase antibodies" OR "anti-thyroglobulin antibodies" OR "thyroid antibodies" OR "tgab" OR "anti-thyroglobulin antibodies" OR "anti-tg" OR "thyroglobulin antibodies" OR "anti-tg antibodies" OR "tg autoantibodies" OR "tpoab" OR "anti-thyroid peroxidase antibodies" OR "anti-tpo" OR "thyroid peroxidase antibodies" OR "anti-tpo antibodies" OR "tpo autoantibodies") AND (db:("LILACS"))

PubMed Results: 78

#1

("diet, gluten free"[MeSH Terms] OR "Gluten-free"[tiab:~0] OR "Gluten deprivation"[tiab:~0] OR "Gluten restriction" [tiab] OR "Gluten withdrawal" [tiab] OR "Gluten elimination" [tiab] OR "Gluten exclusion" [tiab] OR "Gluten avoidance" [tiab] OR "Gluten removal" [tiab])

#2

"thyroid diseases"[MeSH Terms] OR "thyroiditis, autoimmune"[MeSH Terms] OR "Thyroiditis" [tiab] OR "Thyroid inflammation" [tiab] OR "Hashimoto" [tiab] OR "Hashimoto's thyroiditis" [tiab] OR "Hashimoto's disease" [tiab] OR "Chronic lymphocytic thyroiditis" [tiab] OR "Autoimmune thyroiditis" [tiab] OR "Thyroid Disorder" [tiab] OR "Thyroid Disorders" [tiab] OR "Thyroid Disease" [tiab] OR "Thyroid Diseases" [tiab] OR "Thyroid Autoimmunity" [tiab] OR "Autoimmune Thyroid Disease" [tiab] OR "Autoimmune Thyroid Disorders" [tiab] OR "Hashimoto's Thyroiditis" [tiab] OR "Hashimoto's Disease" [tiab] OR "Chronic Thyroiditis" [tiab] OR "Lymphocytic Thyroiditis" [tiab] OR "Autoimmune Thyroiditis" [tiab] OR "Thyroid Gland Inflammation" [tiab] OR "Thyroid Inflammatory Disease" [tiab]

#3

((("clinical trial"[Publication Type] OR "comparative study"[Publication Type] OR ("control"[Text Word] AND "study"[Text Word]) OR "program"[Text Word] OR "epidemiologic studies"[MeSH Terms]) NOT (("animals"[MeSH Terms:noexp] NOT "humans"[MeSH Terms:noexp]) OR "comment"[Publication Type] OR "editorial"[Publication Type] OR "review"[Publication Type] OR "meta analysis"[Publication Type] OR "case report"[Text Word] OR "consensus"[MeSH Terms] OR "guideline"[Publication Type] OR "history"[MeSH Subheading])) OR (("randomized controlled trial"[Publication Type] OR "controlled clinical trial"[Publication Type] OR "randomized"[Title/Abstract] OR "placebo"[Title/Abstract] OR "drug

therapy"[MeSH Subheading] OR ("randomly"[Title/Abstract] OR "trial"[Title/Abstract] OR "groups"[Title/Abstract])) NOT ("animals"[MeSH Terms] NOT "humans"[MeSH Terms])))   #4   #1 AND #2 AND #3
**LILACS (BVS)**   **Results: 1**   ("gluten free diet" OR "gluten-free" OR "gluten deprivation" OR "gluten restriction" OR "gluten withdrawal" OR "gluten elimination" OR "gluten exclusion" OR "gluten avoidance" OR "gluten removal") AND ("thyroid disease" OR "autoimmune thyroiditis" OR "thyroiditis" OR "thyroid inflammation" OR "hashimoto" OR "hashimoto`s thyroiditis" OR "hashimoto`s disease" OR "chronic lymphocytic thyroiditis" OR "autoimmune thyroiditis" OR "tsh" OR "thyroid stimulating hormone" OR "thyrotropin" OR "thyrotropic hormone" OR "tsh hormone" OR "ft4" OR "free thyroxine" OR "free t4" OR "free thyroxine level" OR "serum free t4" OR "free thyroxine concentration" OR "anti-thyroid antibodies" OR "thyroid autoantibodies" OR "anti-thyroid peroxidase antibodies" OR "anti-thyroglobulin antibodies" OR "thyroid antibodies" OR "tgab" OR "anti-thyroglobulin antibodies" OR "anti-tg" OR "thyroglobulin antibodies" OR "anti-tg antibodies" OR "tg autoantibodies" OR "tpoab" OR "anti-thyroid peroxidase antibodies" OR "anti-tpo" OR "thyroid peroxidase antibodies" OR "anti-tpo antibodies" OR "tpo autoantibodies") AND (db:("LILACS")
**SCOPUS (Elsevier) –**   **Results: 92**   #1   TITLE-ABS-KEY("gluten free diet" OR "gluten-free" OR "gluten deprivation" OR "gluten restriction" OR "gluten withdrawal" OR "gluten elimination" OR "gluten exclusion" OR "gluten avoidance" OR "gluten removal")   #2   TITLE-ABS-KEY("thyroid diseases" OR "autoimmune thyroiditis" OR "Thyroiditis" OR "Thyroid inflammation" OR "Hashimoto" OR "Hashimoto's thyroiditis" OR "Hashimoto's disease" OR "Chronic lymphocytic thyroiditis" OR "Autoimmune thyroiditis" OR "Thyroid Disorder" OR "Thyroid Disorders" OR "Thyroid Disease" OR "Thyroid Diseases" OR "Thyroid Autoimmunity" OR "Autoimmune Thyroid Disease" OR "Autoimmune Thyroid Disorders" OR "Chronic Thyroiditis" OR "Lymphocytic Thyroiditis" OR "Autoimmune Thyroiditis" OR "Inflammation of the Thyroid" OR "Thyroid Gland Inflammation" OR "Thyroid Inflammatory Disease" OR "Thyroid Inflammatory Disorders")

#3

TITLE-ABS-KEY "clinical trials" OR "clinical trials as a topic" OR "randomized controlled trial" OR "Randomized Controlled Trials as Topic" OR "controlled clinical trial" OR "Controlled Clinical Trials" OR "random allocation" OR "Double-Blind Method" OR "Single-Blind Method" OR "Cross-Over Studies" OR "Placebos" OR "multicenter study" OR "double blind procedure" OR "single blind procedure" OR "crossover procedure" OR "clinical trial" OR "controlled study" OR "randomization" OR "placebo" OR "clinical trials" OR "clinical trials as a topic" OR "randomized controlled trial" OR "Randomized Controlled Trials as Topic" OR "controlled clinical trial" OR "Controlled Clinical Trials as Topic" OR "random allocation" OR "randomly allocated" OR "allocated randomly" OR "Double-Blind Method" OR "Single-Blind Method" OR "Cross-Over Studies" OR "Placebos" OR "cross-over trial" OR "single blind" OR "double blind" OR "factorial design" OR "factorial trial" OR (clinical AND trial* OR trial* OR rct* OR random* OR blind*)

#4

#1 AND #2 AND #3

Web of Science (Elsevier)-

Results: 181

#1

("gluten free diet" OR "gluten-free" OR "gluten deprivation" OR "gluten restriction" OR "gluten withdrawal" OR "gluten elimination" OR "gluten exclusion" OR "gluten avoidance" OR "gluten removal")

#2

("thyroid diseases" OR "autoimmune thyroiditis" OR "Thyroiditis" OR "Thyroid inflammation" OR "Hashimoto" OR "Hashimoto's thyroiditis" OR "Hashimoto's disease" OR "Chronic lymphocytic thyroiditis" OR "Autoimmune thyroiditis" OR "Thyroid Disorder" OR "Thyroid Disorders" OR "Thyroid Disease" OR "Thyroid Diseases" OR "Thyroid Autoimmunity" OR "Autoimmune Thyroid Disease" OR "Autoimmune Thyroid Disorders" OR "Chronic Thyroiditis" OR "Lymphocytic Thyroiditis" OR "Autoimmune Thyroiditis" OR "Inflammation of the Thyroid" OR "Thyroid Gland Inflammation" OR "Thyroid Inflammatory Disease" OR "Thyroid Inflammatory Disorders")

Gray literature: Proquest (62), Google Scholar (814), Experts (101)

Hashimoto's thyroiditis AND gluten free diet.

("gluten-free" OR "gluten deprivation" OR "gluten restriction" OR "gluten withdrawal" OR "gluten elimination" OR "gluten exclusion" OR "gluten avoidance" OR "gluten removal") AND ("thyroid disease" OR "autoimmune thyroiditis" OR "thyroiditis" OR "thyroid inflammation" OR "hashimoto" OR "hashimoto`s thyroiditis" OR "hashimoto`s disease" OR "chronic lymphocytic thyroiditis" OR "autoimmune thyroiditis"

_Supplementary material S3 - PEER PRESS

PRESS Guideline — Search Submission and Peer Review Assessment

Search Submission: This section is to be filled in by the searcher

Searcher: Andreia Carmo **Email: carmo@unifesp.br**

Date submitted: 15/07/2024 **Date requested by: 15/07/2024**

Search Topic or Title:

EFFECTS OF GLUTEN-FREE DIET INTERVENTION IN THE TREATMENT OF HASHIMOTO'S THYROIDITIS IN NON-CELIAC DISEASE: A SYSTEMATIC REVIEW

This search strategy is:

My PRIMARY (core) database strategy:

☒ This is my first submission ☐ This is submitted after feedback

This search strategy is:

My SECONDARY (supplemental) database strategy:

☐ This is my first submission ☐ This is submitted after feedback

Database(s)

(e.g., MEDLINE, CINAHL, Embase): **[mandatory]**

MEDLINE, EMBASE, Cochrane Library, LILAC's,

Database Platform(s)

(e.g., Ovid, EBSCO): **[mandatory]**

PubMed, Scopus – Elsevier, BVS – LILAC's, Web of Science – Clarivate Analytics e Wiley – Cochrane Library

*If your chosen database or platform provides a link to the search history, please provide it here:

Research Question(s)

(Describe the purpose of the search) **[mandatory]**

Is withdrawing gluten from the diet effective in treating Hashimoto's thyroiditis in non-celiac disease?

PICO(S) or Related Format

(Outline the PICOs, SPIDER, PEPSI, etc. for your question — i.e., **P**atient, **I**ntervention, **C**omparison, **O**utcome, and **S**tudy Design — as applicable)

**P**	Adults and elderly diagnosed with Hashimoto's Thyroiditis and non-celiac disease
**I**	Gluten free diet
**C**	Any gluten dietary intervention; no dietary intervention; placebo (As long as all of them contain gluten*)*

**O**	triiodothyronine fT3), tetraiodothyronine (fT4); Thyroid stimulating hormone (TSH); Peroxidase (TPO) and thyroglobulin (Tg); C-reactive protein (CRP); Vitamin D; Adverse effects; body weight, body mass index (BMI); Diet adherence; Health-related quality of life.
**S**	Randomized controlled trial (RCT's), including cross-over trials

Inclusion Criteria

(List criteria such as age groups, study designs, and so on to be included) [optional]

Adults and elderly diagnosed with HT and non-celiac disease who participate in a Randomized controlled trial (RCT's), including cross-over trials, that follow a gluten free diet intervention. Valid comparator groups will be comprised those receiving any gluten dietary intervention, no dietary intervention or placebo (as long as all of them contain gluten*)*.

Exclusion Criteria

(List criteria such as study designs, date limits, and so on to be excluded) [optional]

(i) pregnant or lactation; (ii) on gluten-reduced diet intervention; (iii) patients with thyroidectomy; (iv) supplementation intervention; (v) interventions with other supporting diets; (vi) multifactorial interventions; (vii) interventions that were designed to induce autoimmune thyroid diseases; (viii) with patients who started HT drug therapy at the same time as GFD; (ix) with patients who changed HT drug therapy during the GFD period; (x) with hyperthyroidism or other endocrine disorders.

Were Search Filters Applied? [mandatory]

☒ Yes ☐ No

If YES, which were used (e.g., Cochrane RCT filter, CADTH's Guidelines filter, PubMed Clinical Queries filter)? Provide the source if this is a published filter. **[mandatory if the answer was YES]**

Filters for clinical trials and comparative studies - Cochrane RCT

Other notes or comments you feel would be useful for the peer reviewer (e.g., decision on date or language limits, articles used in pulling search terms)? **[optional]**

Not applied.

Copy and paste your search strategy here, exactly as run, including the number of hits per line. **[mandatory]**

PubMed
Total Results: 77

#1
("diet, gluten free"[MeSH Terms] OR "Gluten-free"[tiab:~0] OR "Gluten deprivation"[tiab:~0] OR "Gluten restriction" [tiab] OR "Gluten withdrawal" [tiab] OR "Gluten elimination" [tiab] OR "Gluten exclusion" [tiab] OR "Gluten avoidance" [tiab] OR "Gluten removal" [tiab])

#2
"thyroid diseases"[MeSH Terms] OR "thyroiditis, autoimmune"[MeSH Terms] OR "Thyroiditis" [tiab] OR "Thyroid inflammation" [tiab] OR "Hashimoto" [tiab] OR "Hashimoto's thyroiditis" [tiab] OR "Hashimoto's disease" [tiab] OR "Chronic lymphocytic thyroiditis" [tiab] OR "Autoimmune thyroiditis" [tiab] OR "Thyroid Disorder" [tiab] OR "Thyroid Disorders" [tiab] OR "Thyroid Disease" [tiab] OR "Thyroid Diseases" [tiab] OR "Thyroid Autoimmunity" [tiab] OR "Autoimmune Thyroid Disease" [tiab] OR "Autoimmune Thyroid Disorders" [tiab] OR "Hashimoto's Thyroiditis" [tiab] OR "Hashimoto's Disease" [tiab] OR "Chronic Thyroiditis" [tiab] OR "Lymphocytic Thyroiditis" [tiab] OR "Autoimmune Thyroiditis" [tiab] OR "Thyroid Gland Inflammation" [tiab] OR "Thyroid Inflammatory Disease" [tiab]

#**3**
((("clinical trial"[Publication Type] OR "comparative study"[Publication Type] OR ("control"[Text Word] AND "study"[Text Word]) OR "program"[Text Word] OR "epidemiologic studies"[MeSH Terms]) NOT (("animals"[MeSH Terms:noexp] NOT "humans"[MeSH Terms:noexp]) OR "comment"[Publication Type] OR "editorial"[Publication Type] OR "review"[Publication Type] OR "meta analysis"[Publication Type] OR "case report"[Text Word] OR "consensus"[MeSH Terms] OR "guideline"[Publication Type] OR "history"[MeSH Subheading])) OR (("randomized controlled trial"[Publication Type] OR "controlled clinical trial"[Publication Type] OR "randomized"[Title/Abstract] OR "placebo"[Title/Abstract] OR "drug therapy"[MeSH Subheading] OR ("randomly"[Title/Abstract] OR "trial"[Title/Abstract] OR "groups"[Title/Abstract])) NOT ("animals"[MeSH Terms] NOT "humans"[MeSH Terms])))

#4
#1 AND #2 AND #3

EMBASE
Total Results: 77
#1
((('gluten free diet'/exp OR 'gluten-free':ti,ab,kw OR 'gluten deprivation':ti,ab,kw OR 'gluten restriction':ti,ab,kw OR 'gluten withdrawal':ti,ab,kw OR 'gluten elimination':ti,ab,kw OR 'gluten exclusion':ti,ab,kw OR 'gluten avoidance':ti,ab,kw OR 'gluten removal':ti,ab,kw))
#2
(('thyroid disease'/exp OR 'autoimmune thyroiditis'/exp OR 'thyroiditis':ti,ab,kw OR 'thyroid inflammation':ti,ab,kw OR 'hashimoto':ti,ab,kw OR 'hashimoto`s thyroiditis':ti,ab,kw OR 'hashimoto`s disease':ti,ab,kw OR 'chronic lymphocytic thyroiditis':ti,ab,kw OR 'autoimmune thyroiditis':ti,ab,kw OR 'tsh':ti,ab,kw OR 'thyroid stimulating hormone':ti,ab,kw OR 'thyrotropin':ti,ab,kw OR 'thyrotropic hormone':ti,ab,kw OR 'tsh hormone':ti,ab,kw OR 'ft4':ti,ab,kw OR 'free thyroxine':ti,ab,kw OR 'free t4':ti,ab,kw OR 'free thyroxine level':ti,ab,kw OR 'serum free t4':ti,ab,kw OR 'free thyroxine concentration':ti,ab,kw OR 'anti-thyroid antibodies':ti,ab,kw OR 'thyroid autoantibodies':ti,ab,kw OR 'anti-thyroid peroxidase antibodies':ti,ab,kw OR 'anti-thyroglobulin antibodies':ti,ab,kw OR 'thyroid antibodies' OR 'tgab':ti,ab,kw OR 'anti-thyroglobulin antibodies':ti,ab,kw OR 'anti-tg':ti,ab,kw OR 'thyroglobulin antibodies':ti,ab,kw OR 'anti-tg antibodies':ti,ab,kw OR 'tg autoantibodies':ti,ab,kw OR 'tpoab':ti,ab,kw OR 'anti-thyroid peroxidase antibodies':ti,ab,kw OR 'anti-tpo':ti,ab,kw OR 'thyroid peroxidase antibodies':ti,ab,kw OR 'anti-tpo antibodies':ti,ab,kw OR 'tpo autoantibodies':ti,ab,kw)))
#3
'randomized controlled trial'/exp OR 'controlled clinical trial'/de OR random*:ti,ab,tt OR 'randomization'/de OR 'intermethod comparison'/de OR placebo:ti,ab,tt 'double blind procedure'/de OR (parallel NEXT/1 group*):ti,ab,tt OR (crossover:ti,ab,tt OR 'cross over':ti,ab,tt) OR (compare:ti,tt OR compared:ti,tt OR comparison:ti,tt) OR ((evaluated:ab OR evaluate:ab OR evaluating:ab OR assessed:ab OR assess:ab) AND (compare:ab OR compared:ab OR comparing:ab OR comparison:ab)) OR (open NEXT/1 label):ti,ab,tt OR ((double OR single OR doubly OR singly) NEXT/1 (blind OR blinded OR blindly)):ti,ab,tt OR ((assign* OR match OR matched OR allocation) NEAR/6 (alternate OR group OR groups OR intervention OR interventions OR patient OR patients OR subject OR subjects OR participant OR participants)):ti,ab,tt OR (assigned:ti,ab,tt OR allocated:ti,ab,tt) OR (controlled NEAR/8 (study OR design OR trial)):ti,ab,tt OR (volunteer:ti,ab,tt OR volunteers:ti,ab,tt) OR 'human experiment'/de OR trial:ti,tt OR (((random* NEXT/1 sampl* NEAR/8 ('cross section*' OR questionnaire* OR survey OR surveys OR database or databases)):ti,ab,tt) NOT ('comparative study'/de OR 'controlled study'/de OR 'randomised controlled':ti,ab,tt OR 'randomized controlled':ti,ab,tt OR 'randomly assigned':ti,ab,tt)) OR ('cross-sectional study'/de NOT ('randomized controlled trial'/exp OR 'controlled clinical trial'/de OR 'controlled study'/de OR 'randomised controlled':ti,ab,tt OR 'randomized controlled':ti,ab,tt OR 'control group':ti,ab,tt OR 'control groups':ti,ab,tt)) OR ('case control*':ti,ab,tt AND random*:ti,ab,tt NOT ('randomised controlled':ti,ab,tt OR 'randomized controlled':ti,ab,tt)) OR 'random field*':ti,ab,tt OR ('random cluster' NEAR/4 sampl*):ti,ab,tt
#4

#1 AND #2 AND #3

Cochrane Library –
Total Results: 3
#1 ('gluten free diet':ti,ab,kw OR 'gluten-free':ti,ab,kw OR 'gluten deprivation':ti,ab,kw OR 'gluten restriction':ti,ab,kw OR 'gluten withdrawal':ti,ab,kw OR 'gluten elimination':ti,ab,kw OR 'gluten exclusion':ti,ab,kw OR 'gluten avoidance':ti,ab,kw OR 'gluten removal':ti,ab,kw OR
#2 MeSH descriptor: [Diet, Gluten-Free] explode all trees
#3 #1 OR #2
#4 (('thyroid disease':ti,ab,kw OR 'autoimmune thyroiditis':ti,ab,kw OR 'thyroiditis':ti,ab,kw OR 'thyroid inflammation':ti,ab,kw OR 'hashimoto':ti,ab,kw OR 'hashimoto`s thyroiditis':ti,ab,kw OR 'hashimoto`s disease':ti,ab,kw OR 'chronic lymphocytic thyroiditis':ti,ab,kw OR 'autoimmune thyroiditis':ti,ab,kw OR 'tsh':ti,ab,kw OR 'thyroid stimulating hormone':ti,ab,kw OR 'thyrotropin':ti,ab,kw OR 'thyrotropic hormone':ti,ab,kw OR 'tsh hormone':ti,ab,kw OR 'ft4':ti,ab,kw OR 'free thyroxine':ti,ab,kw OR 'free t4':ti,ab,kw OR 'free thyroxine level':ti,ab,kw OR 'serum free t4':ti,ab,kw OR 'free thyroxine concentration':ti,ab,kw OR 'anti-thyroid antibodies':ti,ab,kw OR 'thyroid autoantibodies':ti,ab,kw OR 'anti-thyroid peroxidase antibodies':ti,ab,kw OR 'anti-thyroglobulin antibodies':ti,ab,kw OR 'thyroid antibodies':ti,ab,kw OR 'tgab':ti,ab,kw OR 'anti-thyroglobulin antibodies':ti,ab,kw OR 'anti-tg':ti,ab,kw OR 'thyroglobulin antibodies':ti,ab,kw OR 'anti-tg antibodies':ti,ab,kw OR 'tg autoantibodies':ti,ab,kw OR 'tpoab':ti,ab,kw OR 'anti-thyroid peroxidase antibodies':ti,ab,kw OR 'anti-tpo':ti,ab,kw OR 'thyroid peroxidase antibodies':ti,ab,kw OR 'anti-tpo antibodies':ti,ab,kw OR 'tpo autoantibodies':ti,ab,kw))
#5 MeSH descriptor: [Thyroid Diseases] explode all trees
#6 #5 OR #5

#7 #3 AND #6 3#1 AND #2

SCOPUS –
Total Results: 106
#1
TITLE-ABS-KEY("gluten free diet" OR "gluten-free" OR "gluten deprivation" OR "gluten restriction" OR "gluten withdrawal" OR "gluten elimination" OR "gluten exclusion" OR "gluten avoidance" OR "gluten removal")

#2
TITLE-ABS-KEY("thyroid disease" OR "autoimmune thyroiditis" OR "thyroiditis" OR "thyroid inflammation" OR "hashimoto" OR "hashimoto`s thyroiditis" OR "hashimoto`s disease" OR "chronic lymphocytic thyroiditis" OR "autoimmune thyroiditis" OR "tsh" OR "thyroid stimulating hormone" OR "thyrotropin" OR "thyrotropic hormone" OR "tsh hormone" OR "ft4" OR "free thyroxine" OR "free t4" OR "free thyroxine level" OR "serum free t4" OR "free thyroxine concentration" OR "anti-thyroid antibodies" OR "thyroid autoantibodies" OR "anti-thyroid peroxidase antibodies" OR "anti-thyroglobulin antibodies" OR "thyroid antibodies"

OR "tgab" OR "anti-thyroglobulin antibodies" OR "anti-tg" OR "thyroglobulin antibodies" OR "anti-tg antibodies" OR "tg autoantibodies" OR "tpoab" OR "anti-thyroid peroxidase antibodies" OR "anti-tpo" OR "thyroid peroxidase antibodies" OR "anti-tpo antibodies" OR "tpo autoantibodies")

#3
TITLE-ABS-KEY "clinical trials" OR "clinical trials as a topic" OR "randomized controlled trial" OR "Randomized Controlled Trials as Topic" OR "controlled clinical trial" OR "Controlled Clinical Trials" OR "random allocation" OR "Double-Blind Method" OR "Single-Blind Method" OR "Cross-Over Studies" OR "Placebos" OR "multicenter study" OR "double blind procedure" OR "single blind procedure" OR "crossover procedure" OR "clinical trial" OR "controlled study" OR "randomization" OR "placebo" OR "clinical trials" OR "clinical trials as a topic" OR "randomized controlled trial" OR "Randomized Controlled Trials as Topic" OR "controlled clinical trial" OR "Controlled Clinical Trials as Topic" OR "random allocation" OR "randomly allocated" OR "allocated randomly" OR "Double-Blind Method" OR "Single-Blind Method" OR "Cross-Over Studies" OR "Placebos" OR "cross-over trial" OR "single blind" OR "double blind" OR "factorial design" OR "factorial trial" OR "factorial trial" OR (clinical AND trial* OR trial* OR rct* OR random* OR blind*)

#**4**
#1 AND #2 AND #3

LILAC's-

Total Results: 1

("gluten free diet" OR "gluten-free" OR "gluten deprivation" OR "gluten restriction" OR "gluten withdrawal" OR "gluten elimination" OR "gluten exclusion" OR "gluten avoidance" OR "gluten removal") AND ("thyroid disease" OR "autoimmune thyroiditis" OR "thyroiditis" OR "thyroid inflammation" OR "hashimoto" OR "hashimoto`s thyroiditis" OR "hashimoto`s disease" OR "chronic lymphocytic thyroiditis" OR "autoimmune thyroiditis" OR "tsh" OR "thyroid stimulating hormone" OR "thyrotropin" OR "thyrotropic hormone" OR "tsh hormone" OR "ft4" OR "free thyroxine" OR "free t4" OR "free thyroxine level" OR "serum free t4" OR "free thyroxine concentration" OR "anti-thyroid antibodies" OR "thyroid autoantibodies" OR "anti-thyroid peroxidase antibodies" OR "anti-thyroglobulin antibodies" OR "thyroid antibodies" OR "tgab" OR "anti-thyroglobulin antibodies" OR "anti-tg" OR "thyroglobulin antibodies" OR "anti-tg antibodies" OR "tg autoantibodies" OR "tpoab" OR "anti-thyroid peroxidase antibodies" OR "anti-tpo" OR "thyroid peroxidase antibodies" OR "anti-tpo antibodies" OR "tpo autoantibodies") AND (db:("LILACS"))

Web of Science –

Total Results: 159

#1

("gluten free diet" OR "gluten-free" OR "gluten deprivation" OR "gluten restriction" OR "gluten withdrawal" OR "gluten elimination" OR "gluten exclusion" OR "gluten avoidance" OR "gluten removal")

#2

("thyroid disease" OR "autoimmune thyroiditis" OR "thyroiditis" OR "thyroid inflammation" OR "hashimoto" OR "hashimoto`s thyroiditis" OR "hashimoto`s disease" OR "chronic lymphocytic thyroiditis" OR "autoimmune thyroiditis" OR "tsh" OR "thyroid stimulating hormone" OR "thyrotropin" OR "thyrotropic hormone" OR "tsh hormone" OR "ft4" OR "free thyroxine" OR "free t4" OR "free thyroxine level" OR "serum free t4" OR "free thyroxine concentration" OR "anti-thyroid antibodies" OR "thyroid autoantibodies" OR "anti-thyroid peroxidase antibodies" OR "anti-thyroglobulin antibodies" OR "thyroid antibodies" OR "tgfb" OR "anti-thyroglobulin antibodies" OR "anti-tg" OR "thyroglobulin antibodies" OR "anti-tg antibodies" OR "tg autoantibodies" OR "tpoat" OR "anti-thyroid peroxidase antibodies" OR "anti-tpo" OR "thyroid peroxidase antibodies" OR "anti-tpo antibodies" OR "tpo autoantibodies")

Peer Review Assessment: This section is to be filled in by the reviewer

Reviewer: Elaine Hipolito dos Santos Costa de Almeida **Email: elaine.hipolito@unifesp.br**

Date completed: 15/07/2024

1. Translation of Research Question(s)

☒ A) No revisions

☐ B) Revision(s) suggested

☐ C) Revision(s) required

If "B" or "C," please provide an explanation or example:

Click or tap here to enter text.

2. Boolean and Proximity Operators

☒ A) No revisions

☐ B) Revision(s) suggested

☐ C) Revision(s) required

If "B" or "C," please provide an explanation or example:

Click or tap here to enter text.

3. Subject Headings

☒ A) No revisions

☐ B) Revision(s) suggested

☐ C) Revision(s) required

If "B" or "C," please provide an explanation or example:

Click or tap here to enter text.

4. Text Word Searching

☒ A) No revisions

☐ B) Revision(s) suggested

☐ C) Revision(s) required

If “B” or “C,” please provide an explanation or example:

Click or tap here to enter text.

5. Spelling, Syntax, and Line Numbers

☒ A) No revisions

☐ B) Revision(s) suggested

☐ C) Revision(s) required

If “B” or “C,” please provide an explanation or example:

Click or tap here to enter text.

6. Limits and Filters

☒ A) No revisions

☐ B) Revision(s) suggested

☐ C) Revision(s) required

If “B” or “C,” please provide an explanation or example:

Click or tap here to enter text.

Overall Evaluation (Note: If 1 or more of the previous elements were “revision required,” this response must be “revisions required.”).

☒ A) No revisions

☐ B) Revision(s) suggested

☐ C) Revision(s) required

Additional Comments (including sources of additional search terms, such as the use of text mining software or resources such as ChemID):

Click or tap here to enter text.

Please select the most appropriate answer for each element.

Element	No revisions	Revision(s) suggested	Revision(s) required
1. Translation of research question	☒	☐	☐
2. Boolean and proximity operators	☒	☐	☐
3. Subject headings	☒	☐	☐
4. Text word searching	☒	☐	☐
5. Spelling, syntax, and line numbers	☒	☐	☐
6. Limits and filters	☒	☐	☐
**Overall evaluation**	☒	☐	☐

If revisions are suggested or required, please provide an explanation or example:

Click or tap here to enter text.

__Supplementary material S4 - Characteristics of excluded studies: databases and other methods

S4A - Characteristics of excluded studies (databases and registers)

Author, Year	Reason for Exclusion*
1 Aslan ES et al, 2024	1
2 Basturk Berrak, 2024 (NCT06419309)	2
3 Godhwani A, Lo H, Ruiz C, 2023**	3
4 Ihnatowicz P, Gębski J, Drywień ME, 2023	3
5 Krysiak R, Kowalcze K, Okopień B, 2022	3
6 Krysiak R, Szkróbka W, Okopień B, 2019	3
7 Ostrowska L, Gier D, Zyśk B, 2021	1
8Riseh SH et al., 2016	3
9 Szczuko M, et al., 2024	3

* (1) Supplementation intervention or interventions with other supporting diets; (2) Ongoing study; (3) non-randomized clinical trial.

**The full article was unavailable, and we consulted the author, who informed us that it was not a randomized clinical article.

References

1 Aslan E S, Meral G, Aydin E, et al. The Effect of a Casein and Gluten-Free Diet on the Epigenetic Characteristics of FoxP3 in Patients with Hashimoto's Thyroiditis. Cureus. (June 26, 2024), 16(6): e63208. DOI:10.7759/cureus.63208

2 Basturk, Berrak. Comparison of the Effect of Gluten-Free-Lactose-Free / Aronia Melanocarpa Supplemented Diet in Patients with Hashimoto's Thyroiditis. Halic University, Istanbul/Turkey, 2024, NCT06419309 (Ongoing study).

3 Godhwani A, Lo H, Ruiz C. Does a Gluten-Free Diet Reduce Symptoms of Autoimmune Thyroid Disease? American Family Physician. 2023 Jul;108(1):87-88. PMID: 37440746

4 Ihnatowicz P, Gębski J, Drywień ME. Efeitos da dieta do Protocolo Autoimune (AIP) nas alterações dos parâmetros da tireoide na doença de Hashimoto. Ann Agric Environ Med. 2023;30(3):513-21. DOI: https://doi.org/10.26444/aaem/166263.

5 Krysiak R, Kowalcze K, Okopień B. Gluten-free diet attenuates the impact of exogenous vitamin D on thyroid autoimmunity in young women with autoimmune thyroiditis: a pilot study. Scand J Clin Lab Invest. 2022;82(7-8):518-524. DOI:10.1080/00365513.2022.2129434

6 Krysiak R, Szkróbka W, Okopień B. The Effect of Gluten-Free Diet on Thyroid Autoimmunity in Drug-Naïve Women with Hashimoto's Thyroiditis: A Pilot Study. Exp Clin Endocrinol Diabetes 2019; 127(07): 417-422. DOI: 10.1055/a-0653-7108

7 Ostrowska L, Gier D, Zyśk B. The Influence of Reducing Diets on Changes in Thyroid Parameters in Women Suffering from Obesity and Hashimoto's Disease. *Nutrients*. 2021; 13(3):862. DOI: https://doi.org/10.3390/nu13030862

8 Riseh SH, Mobasseri M, Jafarabadi M-A, Abbasalizad Farhangi M, Ajorlou E. Nutritional intakes, lipid profile and serum apo-lipoproteins concentrations and their relationship with antithyroid, antigliadin and anti-tissue transglutaminase antibodies in patients with Hashimoto's thyroiditis. Progr Nutr [Internet]. 2016 Dec. 22 [cited 2024 Sep. 20];18(4):376-84. Available from: https://www.mattioli1885journals.com/index.php/progressinnutrition/article/view/4418

9 Szczuko M, Kacprzak J, Przybylska A, Szczuko U, Pobłocki J, Syrenicz A, and Malgorzata AD: The Influence of an Anti-Inflammatory Gluten-Free Diet with EPA and DHA on the Involvement of Maresin and Resolvins in Hashimoto's Disease. Int. J. Mol. Sci. 2024, 25, 11692. https://doi.org/10.3390/ijms252111692. Available from: https://pmc.ncbi.nlm.nih.gov/articles/PMC11546266/pdf/ijms-25-11692.pdf

S4B - Characteristics of excluded studies (other methods)

Author, Year	Reason for Exclusion*
1 Abbott RD, Sadowski A, Alt AG, 2019	1
2 Di Sabatino et al., 2015	3
3 Krysiak R, Szkróbka W, Okopień B, 2019	1
4 Krysiak, Kowalcze, Okopień, 2022	1
5 Pobłocki J et al, 2021	4
6 Rodziewicz A, Szewczyk A, Bryl E, 2024	4
7 Ülker MT, Çolak GA, Baş M, 2023	4

1. Non-randomized clinical trial; 2. Supplementation intervention or interventions with other supporting diets; 3. Hashimoto's thyroiditis not reported; 4. Duplicated.

References

1 Abbott R D, Sadowski A, Alt A G. Efficacy of the Autoimmune Protocol Diet as Part of a Multi-disciplinary, Supported Lifestyle Intervention for Hashimoto's Thyroiditis. Cureus. (April 27, 2019). 11(4): e4556. DOI:10.7759/cureus.4556

2 Di Sabatino A, et al. Small Amounts of Gluten in Subjects With Suspected Nonceliac Gluten Sensitivity: A Randomized, Double-Blind, Placebo-Controlled, Cross-Over Trial. Clinical Gastroenterology and Hepatology. 2015, 13(9):1604-1612.e3. DOI: https://doi.org/10.1016/j.cgh.2015.01.029

3 Krysiak R, Kowalcze K, Okopień B. Gluten-free diet attenuates the impact of exogenous vitamin D on thyroid autoimmunity in young women with autoimmune thyroiditis: a pilot study. Scand J Clin Lab Invest. 2022;82(7-8):518-524. DOI:10.1080/00365513.2022.2129434

4 Krysiak R, Szkróbka W, Okopień B. The Effect of Gluten-Free Diet on Thyroid Autoimmunity in Drug-Naïve Women with Hashimoto's Thyroiditis: A Pilot Study. Exp Clin Endocrinol Diabetes 2019; 127(07): 417-422. DOI: 10.1055/a-0653-7108

5 Pobłocki J, Pańka T, Szczuko M, Telesiński A, and Syrenicz A. Whether a Gluten-Free Diet Should Be Recommended in Chronic Autoimmune Thyroiditis or Not? — A 12-Month Follow-Up. J Clin Med. 2021 Aug; 10(15): 3240. Doi: 10.3390/jcm10153240

6 Rodziewicz A, Szewczyk A, Bryl E. Gluten-Free Diet Alters the Gut Microbiome in Women with Autoimmune Thyroiditis. Nutrients. 2024 Mar 1;16(5):1-18.

7 Ülker MT, Çolak GA, Baş M. Evaluation of the effect of gluten-free diet and Mediterranean diet on autoimmune system in patients with Hashimoto's thyroiditis. Food Sci Nutr. 2023 Nov 20;12(2):1180-1188. Available from: https://onlinelibrary.wiley.com/doi/10.1002/fsn3.3833.

__Supplementary material S5 - Study characteristics of the published studies included in this systematic review.

ID	Authors	Year	Country	n	Study Design	Sex	Age means (SD or CI)	Some eligibility criteria	Adverse effects	Funding
No Information	Lagana M and Piticchio T et al. (18)	2025	Italy	30 (10M. 20F)	RCT, single-blind study	Men/women	43,25* (11,858**) GFD; 42,25* (12,434**) FD	Inclusion: HT diagnosed (positive TPO-Ab and/or Tg-Ab or ultrasonography image typical; BMI (18.5–29.5kg/m2); stable eating habits in the previous six months. Exclusion: obesity > 30 kg/m2; diabetes, kidney failure, neoplastic and inflammatory diseases; smoking history and alcohol abuse. None of them were taking L-thyroxine	Not evaluated	No external funding

No information	Poblocki J et al {Pobłocki, 2021 #4}(64)	2021	Poland	62	RCT	Women	36.64 (33.66-39.63) GFD; 37.07 (33.83-40.31) CG	Inclusion: HT was diagnosed based on the ultrasound image, which typically shows elevated blood serum levels of anti-TPO and/or anti-Tg. Women on thyroid medication. Exclusion: GFD or periodic elimination of gluten from the diet.	Not evaluated	No external funding
NCT06249074	Rodziewicz L, Szewczyk D, Bryl E (65)	2024	Poland	28	RCT, double-blind study	Women	34.6 (6.3) GG; 36.6 (7.3) PG	Inclusion: thyroid hormones within a normal range and BMI (18.5–24.9 kg/m2). Women on thyroid medication. Exclusion: newly diagnosed AIT and unregulated thyroid hormones gluten-free diet followed during 6 months prior to enrollment	Unfavorable changes in microbiota	Nutricia Foundation and Medical University

NCT05949671	Ulker M et al (56)	2023	Turkey	20	RCT, single-blind study	Women	39.05 (7.52)	Inclusion: BMI between 18.5 kg/m2 and 30 kg/m2. Exclusion: women on thyroid function medication, following an autoimmune diet, with other diagnosed autoimmune diseases, and pregnant or breastfeeding women	Not evaluated	No declaration

GG= Gluten Group; GFD= Gluten Free Diet PG= Placebo Group= Control Group (CG); CD= Celiac Disease; EmA: anti-endomysial antibodies. tTG= anti-tissue transglutaminase antibodies; TPO= Anti-thyroid Peroxidase; CRP= C-reactive protein. Ig: immunoglobulin; AGA: anti-gliadin antibodies; Cap= capsules; BMI= Body Mass Index; HT= Hashimoto`s thyroiditis. SD= Standard deviation; CI= confidence interval. M= Male; F= Female. RCT= Randomized clinical trial.

*Estimated mean; **estimated standard deviation.

***Non-meta-analyzed study.

Only the information for this systematic review was collected (PICOS).

__Supplementary material S6 - Certainty of evidence (GRADE)

Gluten-free diet compared to gluten diet for patients with Hashimoto's thyroiditis without celiac disease.

Autor(es): Edilene Maria Queiroz Araújo; Claubert Radamés Coutinho-Lima; Lana Mércia Santiago de Souza; Andre Silva de Sousa; Helton Estrela Ramos; Bianca de Almeida-Pititto; Graziela De Luca Canto; Virginia Fernandes Moça Trevisani.

Bibliografia: Schunemann, Holger et al., 2022.

Certainty assessment							Summary of findings				
Participants (studies) Follow-up	Risk of bias	Inconsistency	Indirectness	Imprecision	Publication bias	Overall certainty of evidence	Study event rates (%)		Relative effect (95% CI)	Anticipated absolute effects	
							With dieta com glúten	With Dieta sem glúten		Risk with dieta com glúten	Risk difference with Dieta sem glúten
**Thyroid stimulating hormone (follow-up: mean 3 months; assessed with: µIU/mL)**											
110 (3 RCTs)	serious[a]	not serious	not serious	extremely serious[b]	none	⊕◯◯◯ Very low[a,b]	54	56	-	54	MD **0.63 µIU/mL lower** (1.63 lower to 0.36 higher)
**Triiodothyronine (follow-up: mean 3 months; assessed with: pg/mL)**											
110 (3 RCTs)	serious[a]	not serious	not serious	extremely serious[c]	none	⊕◯◯◯ Very low[a,c]	54	56	-	54	MD **0.18 pg/mL lower** (0.5 higher to 0.14 higher)
**Tetraiodothyronine (follow-up: mean 3 months; assessed with: ng/dL)**											
110 (3 RCTs)	serious[d]	not serious	not serious	extremely serious[e]	none	⊕◯◯◯ Very low[d,e]	54	56	-	54	MD **0.33 ng/dL lower** (0.89 lower to 0.23 higher)
**Anti-thyroid Peroxidase (follow-up: mean 3 months; assessed with: IU/mL)**											
110 (3 RCTs)	serious[d]	not serious	not serious	extremely serious[f]	none	⊕◯◯◯ Very low[d,f]	54	56	-	54	MD **76.19 IU/mL higher** (43.86 higher to 108.51 higher)
**Anti-thyroglobulin (follow-up: mean 3 months; assessed with: IU/mL)**											
110 (3 RCTs)	serious[d]	not serious	not serious	extremely serious[g]	none	⊕◯◯◯ Very low[d,g]	54	56	-	54	MD **10.07 IU/mL lower** (17.73 lower to 2.42 lower)
**C-reactive protein (follow-up: mean 4 weeks; assessed with: mg/dL)**											
28 (1 RCT)	not serious	not serious	not serious	extremely serious[h]	none	⊕◯◯◯ Very low[h]					
**Body weight (follow-up: mean 3 months; assessed with: Kg)**											
20 (1 RCT)	serious[d]	not serious	not serious	extremely serious[i]	none	⊕◯◯◯ Very low[d,i]					
**Body Mass Index (follow-up: mean 3 months; assessed with: Kg/m2)**											
20 (1 RCT)	serious[d]	not serious	not serious	extremely serious[j]	none	⊕◯◯◯ Very low[d,j]					

CI: confidence interval; **MD:** mean difference

Explanations:

a. We downgraded one level due to methodological limitations: 2 studies are at high risk of bias overall (Ulker et al. and Poblocki et al.), with some concerns in the following areas: randomization process, deviations from the intended interventions, and incomplete data.

b. We downgraded two levels (3 levels are impossible) due to extremely serious imprecision: the CI ranges from a large beneficial effect to a small harmful effect (SMD -0.94 μIU/mL, 95% CI -2.25, 0.38].

c. We downgraded two levels (three levels are impossible) due to extremely serious imprecision: the CI ranges from a large beneficial effect to a small harmful effect (SMD -0.60 pg/mL, 95% CI -1.64, 0.45).

d. We downgraded one level due to methodological limitations: Two studies were at high risk of bias overall (Ulker et al. and Poblocki et al.), with some concerns in the following domains: randomization process, deviations from the intended interventions and incomplete data. There was still a high risk of bias (Ulker et al. article).

e. We downgraded two levels (3 levels are not possible) due to extremely serious imprecision: the CI ranges from a large beneficial effect to a harmful moderate impact (SMD -0.78 ng/dL, 95% CI -2.16, 0.59).

f. We downgraded two levels (3 are impossible) due to extremely serious imprecision: the CI ranges from trivial/no effect to a large detrimental effect (SMD 0.78 ng/dL, 95% CI -2.16, 0.0). effect (SMD 0.79 IU/mL, 95% CI -0.09, 1.68).

g. We downgraded two levels (3 levels are not possible) due to extremely serious imprecision: the CI ranges from a large beneficial effect to a trivial effect or no effect (SMD -0.39 IU/mL, 95% CI -0.84, 0.06)

h. We downgraded three levels: The CI ranges from a large beneficial effect to a small harmful effect (SMD -0.49mg/dL, 95% CI -1.25, 0.26).

i. We downgraded two levels (3 are impossible) due to extremely serious imprecision: the CI ranges from a large beneficial effect to a moderate harmful effect (SMD -0.23 kg, 95% CI -1.11, 0.65).

j. We downgraded two levels (3 levels are not possible) due to extremely serious imprecision: the CI ranges from a large beneficial effect to a trivial effect or no effect (SMD -1.01 Kg/m2, 95% CI -1.96, -0.07).
